# Supplementary material for: A gas breathing hydrogen/air biofuel cell comprising a redox polymer/hydrogenase-based bioanode
Source: Nat Commun. 2018 Nov 9;9:4715. doi: 10.1038/s41467-018-07137-6 (PMC6226449; doi:10.1038/s41467-018-07137-6)
Supplement: Supplementary file 1 — Supplementary Information [file 41467_2018_7137_MOESM1_ESM.pdf]

Supplementary Information

A gas breathing hydrogen/air biofuel cell comprising a redox polymer/hydrogenase-based bioanode

Szczesny et al.

Supplementary Figures and Schemes

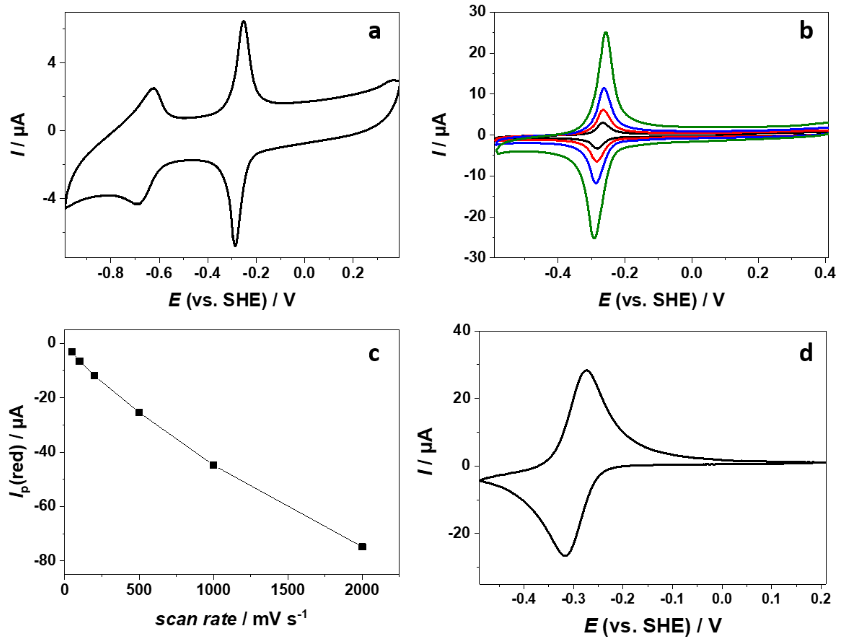

Supplementary Figure 1: Cyclic voltammograms (**a** and **b**) and peak currents of the first reduction (**c**) in 0.1 M KCl/water and cyclic voltammograms in 0.1 M PB at pH 7.4 (**d**) of a P(GMA-BA-PEGMA)-vio film deposited onto a glassy carbon electrode (drop-cast process from acetone). For results shown in **a-c** the PF_6^- salt of the polymer was used; for **c** the purified polymer bearing Cl^- counterions was used. **a**: potential cycle over the first and second reversible reduction of the viologen units at a scan rate of 100 mV s^{-1}. The mid-point potential of the first (E_1) and second (E_2) reduction were derived to be E_1 = -0.27 and E_2 = -0.66 V vs. SHE. **b**: Potential cycles over the first reduction wave measured with different scan rates of 50 (black trace), 100 (red trace), 200 (blue trace) and 1000 (green trace) mV s^{-1}. **c**: Peak current of the reduction wave (I_p(red)) vs. the scan rate follows roughly a linear trend as expected for a surface confined species and the peak potential separation increases from 18 mV to 61 mV with increasing the scan rate from 50 mV s^{-1} to 2000 mV s^{-1}. Black line is guidance for eyes only. **d**: Cyclic voltammogram of the purified Cl^- salt of the polymer, scan rate 10 mV s^{-1}, 0.1 M PB (pH 7.4); the mid-point potential of the first reduction was derived to be E_1 = -0.30 V vs. SHE and is slightly shifted to more negative values in this buffer system as compared to an aqueous KCl solution (see **a** and **b**).

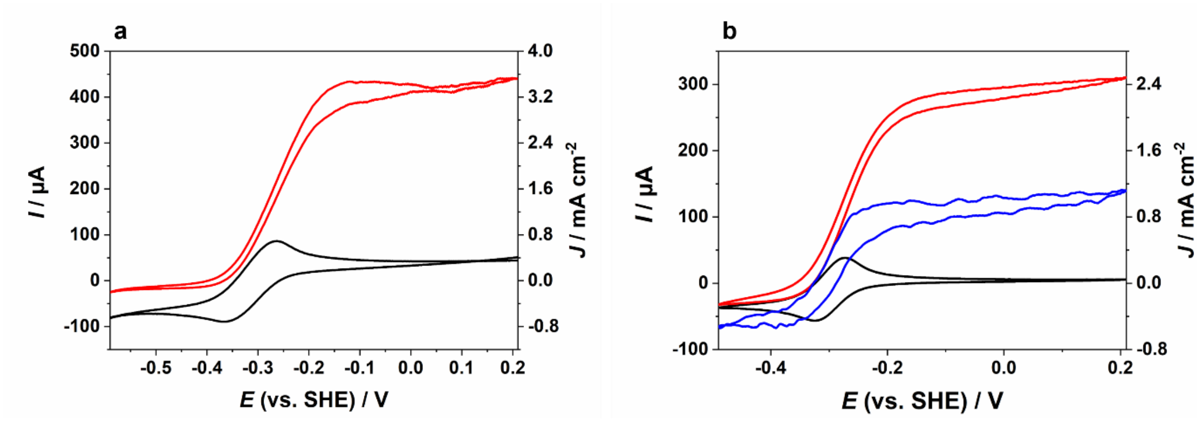

Supplementary Figure 2: Cyclic voltammograms in 0.1 M PB (pH 7.4) recorded with a P(GMA-BA-PEGMA)-vio//P(N_3MA-BA-GMA)-vio/*Dv*H-[NiFeSe] (**a**) and a P(GMA-BA-PEGMA)-vio//P(N_3MA-BA-GMA)-vio/*Dv*MF-[NiFe] (**b**) modified bioanode at scan rates of 10 m V s^{-1} (**a**) and 2 mV s^{-1} (**b**) under argon (black lines) and H_2 atmosphere (blue and red lines). When the substrate H_2 (100 %) is offered in gas breathing mode (red line in **b**) the observed catalytic currents are much higher than the corresponding currents obtained for measurements when H_2 (100 %) is purged through the solution (blue line in **b**). These results demonstrate that indeed a higher local concentration of the substrate was achieved in the gas breathing mode. Nominal hydrogenase loading (absolute value per electrode/normalized to area of bioanode): **a**: 1.53 nmol/12.1 nmol cm^{-2}, **b**: 1.8 mmol/14.3 nmol cm^{-2}.

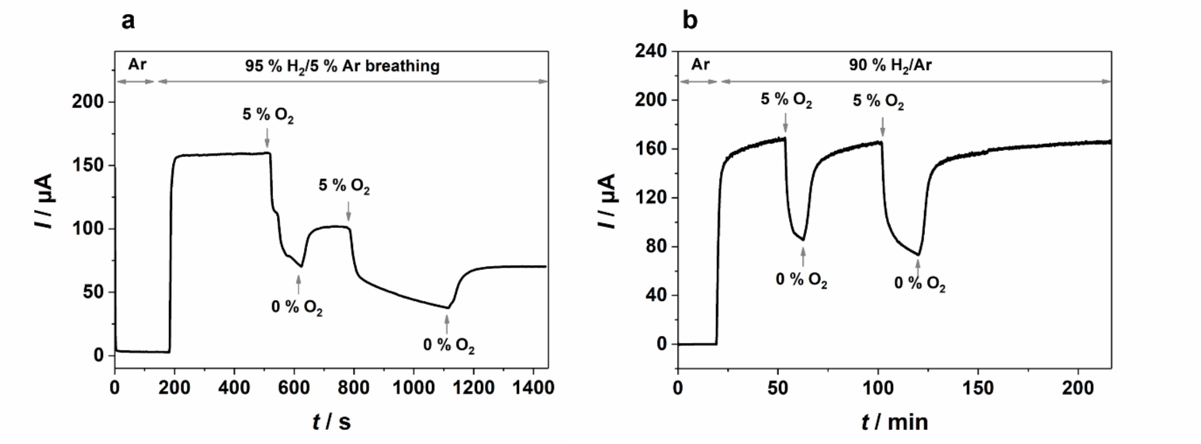

Supplementary Figure 3: Protection from O_2 for a P(GMA-BA-PEGMA)-vio//P(GMA-BA-PEGMA)-vio/*Dv*MF-[NiFe] bioanode. **a**: Chronoamperometry in H_2 breathing configuration (95 % H_2 and 5 % Ar) with O_2 provided from the back together with the substrate (95 % H_2 and 5 % O_2). To maintain inert conditions also in the cell, argon was constantly bubbled through the electrolyte. Obviously, in this case the local O_2 concentration exceeds the protection capacity of the polymer/hydrogenase layer. However, we want to emphasize that in a fuel cell, O_2 is expected to reach the bioanode only from the electrolyte side and in this case, protection is sufficient (see **a** and Figure 3a). **b**: Chronoamperometry of a bioanode that was completely immersed into the electrolyte. Gas mixtures (for composition see graph) were purged through the electrolyte. The bioanode shows the expected behaviour in analogy to polymer/hydrogenase films on flat glassy carbon electrodes[1–3]. The large current drop under aerobic conditions may be an effect of a high O_2 concentration that is reached in the polymer layer since oxygen can penetrate the polymer/enzyme film from the front and the back and thus a fast deactivation of biocatalyst. However, the full recovery of the current is striking and may not only be related to O_2 reduction by the viologen species but also by reactivation of the enzyme by the low potential polymer matrix[1]. Applied potential: +0.16 V vs Ag/AgCl/3 M KCl; electrolyte: 0.1 M PB, pH 7.4. The argon content in the gas feed was adjusted to reach the desired O_2 content. Nominal hydrogenase loading (absolute value per electrode/normalized to area of bioanode): 0.6 nmol/4.8 nmol cm^{-2}.

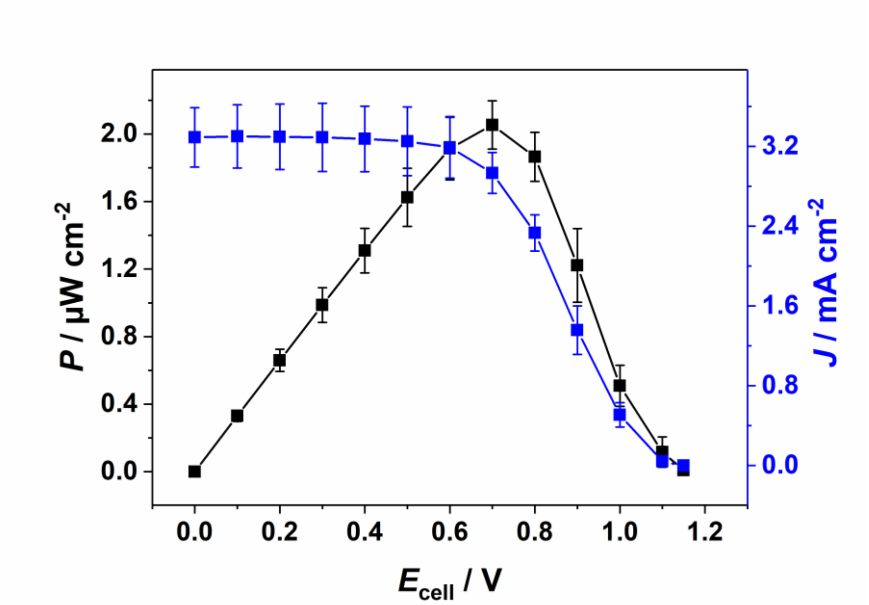

Supplementary Figure 4: Power curve of a biofuel cell comprising a P(GMA-BA-PEGMA)-vio//P(N_3MA-BA-GMA)-vio/*Dv*MF-[NiFe] bioanode and *Mv*-BOx biocathode measured in gas breathing configuration. Power density and current density are shown calculated with respect to the area of the limiting bioanode. For absolute values see Figure 3 in the main text. nominal hydrogenase loading: 1.8 nmol/14.3 nmol cm^{-2}.

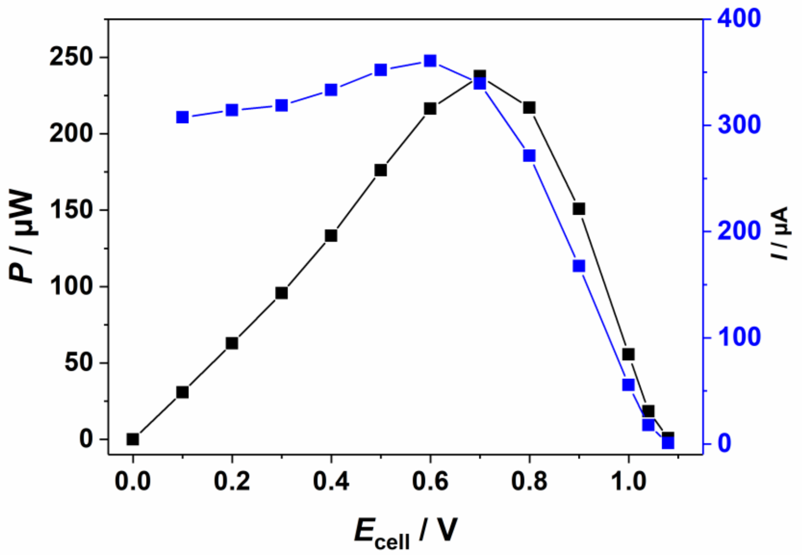

Supplementary Figure 5: Power curve of a biofuel cell comprising a P(GMA-BA-PEGMA)-vio//P(N_3MA-BA-GMA)-vio/*Dv*H-[NiFeSe] bioanode and *Mv*-Box biocathode measured in gas breathing configuration. The OCV of the cell was determined to be 1.08 V and the maximum power output of the cell was 237 µW (or 1.9 mW cm^{-2}) at 0.7 V. Nominal hydrogenase loading: 1.53 nmol/12.1 nmol cm^{-2}.

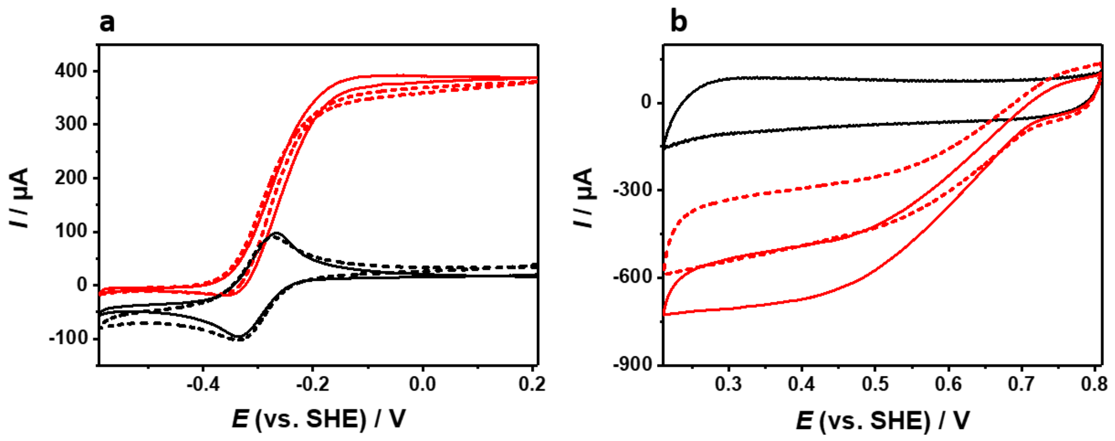

Supplementary Figure 6: Representative cyclic voltammograms recorded before (solid lines) and after (dashed lines) a biofuel cell test with a P(GMA-BA-PEGMA)-vio//P(N_3MA-BA-GMA)-vio/*Dv*MF-[NiFe] bioanode (**a**) and *Mv*-BOx biocathode (**b**) in gas breathing mode under turnover (red lines, **a**: H_2, **b**: air) and non-turnover (black lines, argon) conditions. All voltammograms were measured in 0.1 M PB (pH 7.4) with a scan rate of 10 mV s^{-1}. The biocathode shows a slight decrease of activity after the BFC test, however, the absolute currents are still higher than those measured at the bioanode. Hence, the bioanode remains the limiting electrode throughout the entire BFC evaluation. Nominal hydrogenase loading (absolute value per electrode/normalized to area of bioanode): 1.8 nmol/14.3 nmol cm^{-2}.

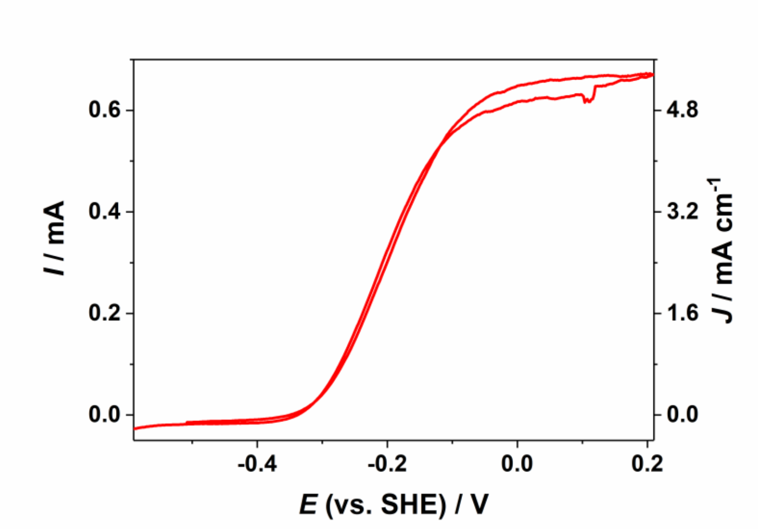

Supplementary Figure 7: Cyclic voltammogram of a high-current density P(GMA-BA-PEGMA)-vio//P(N_3MA-BA-GMA)-vio/*Dv*H-[NiFeSe] bioanode measured in 0.1 M PB (pH 7.4) under H_2 atmosphere. Scan rate: 10 mV s^{-1}. Left ordinate: absolute current *I*; right ordinate: current density *J*. For the calculation of *J* the spot size of the polymer layer was used, which was determined to be ≈4 mm in diameter. Nominal hydrogenase loading: 3.4 nmol/27.0 nmol cm^{-2}.

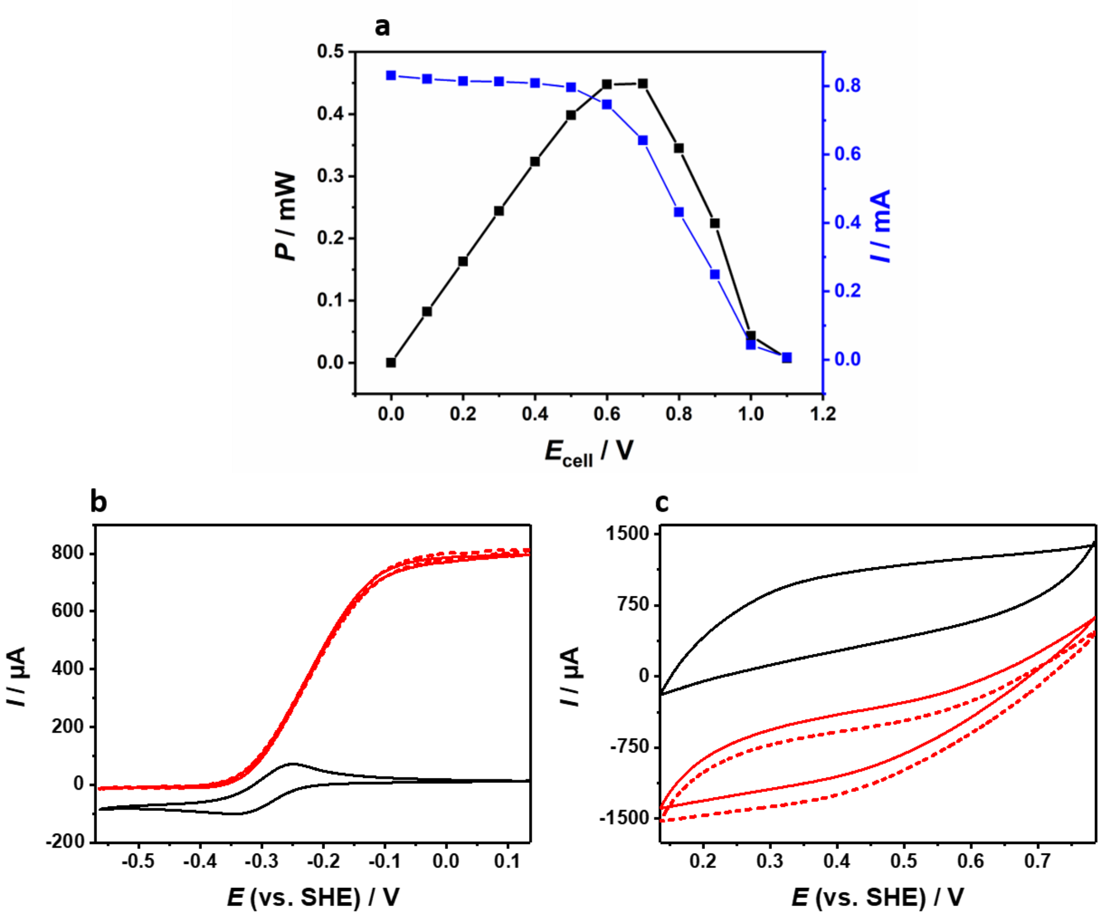

Supplementary Figure 8: Characterization of the high-current density P(GMA-BA-PEGMA)-vio//P(N_3MA-BA-GMA)-vio/*Dv*MF-[NiFe] based BFC (**a**) in the gas breathing mode and the *Mv*-BOx-based biocathode and cyclic voltammograms of the corresponding bioanode (**b**) and biocathode (**c**) recorded before (solid lines) and after (dashed lines) the biofuel cell performance test under turnover (red lines, **b**: H_2, **c**: air) and non-turnover (black lines, argon) conditions. Abolute values are shown. The power curve depicted in A corresponds to the was used for calculation of the normalized current and power values depicted in Figure 4B in the main text. All voltammograms were measured in 0.1 M PB (pH 7.4) with a scan rate of 10 mV s^{-1} and in gas breathing mode. Nominal hydrogenase loading: 4 nmol/31.8 nmol cm^{-2}.

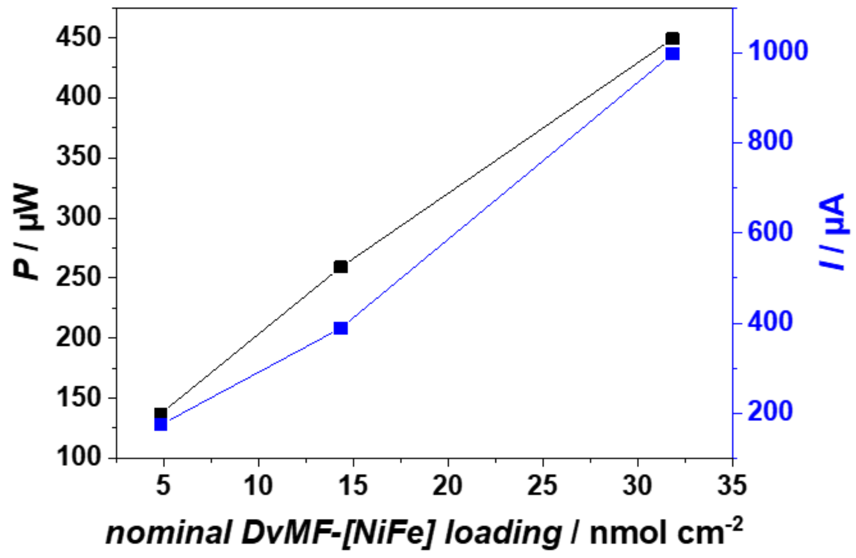

Supplementary Figure 9: Dependency of the absolute current (blue, right ordinate) and absolute power (black, left ordinate, at 0.7 V) values of a P(GMA-BA-PEGMA)-vio//P(N_3MA-BA-GMA)-vio/*Dv*MF-[NiFe] based bioanode (blue, 100 % H_2) and a P(GMA-BA-PEGMA)-vio//P(N_3MA-BA-GMA)-vio/*Dv*MF-[NiFe] based biofuel cell (black, gas breathing mode), respectively. The almost linear increase of I and P indicate that the permanency of the system mainly depends on the amount of deposited catalyst.

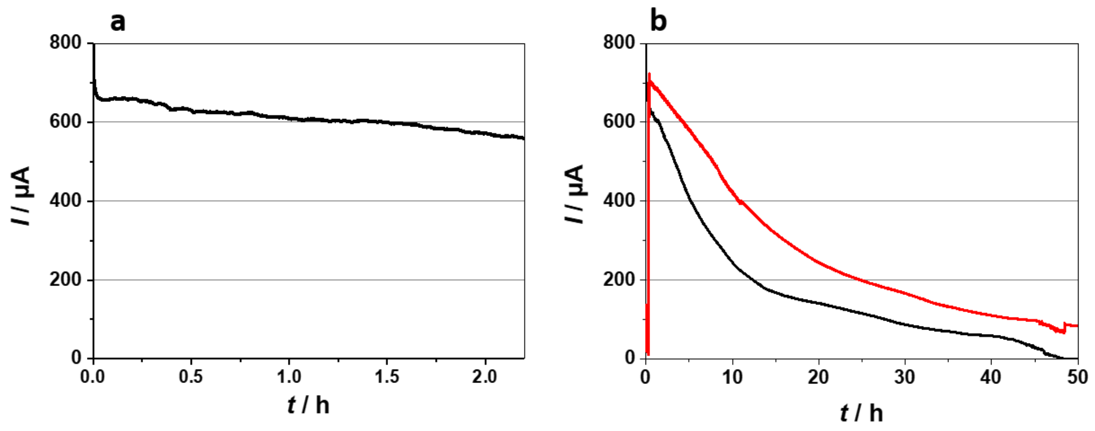

Supplementary Figure 10: Evaluation of the stability of the high-current density *Dv*MF-[NiFe] based biofuel cell at a cell voltage of 0.7 V in 0.1 M PB (pH 7.4) in gas breathing mode. **a**: *t* = 0 – 2.2 h; **b**: 0 – 50 h; black lines in **a** and **b**: current of the biofuel cell at 0.7 V cell voltage, red line in **b**: current of the single *Dv*MF-[NiFe] bioanode at an applied potential of +0.16 V vs. SHE. The course of the current decay for the biofuel cell and the bioanode are similar. The lifetime of the biofuel cell seems to be determined by the degradation of the high-current density polymer/hydrogenase bioanode. Nominal hydrogenase loading: 4 nmol/31.8 nmol cm^{-2}. The pH value of the bulk solution is still neutral after biofuel cell evaluation. However, a local pH change at or in the pores of the electrode cannot be excluded and may contribute to deactivation.

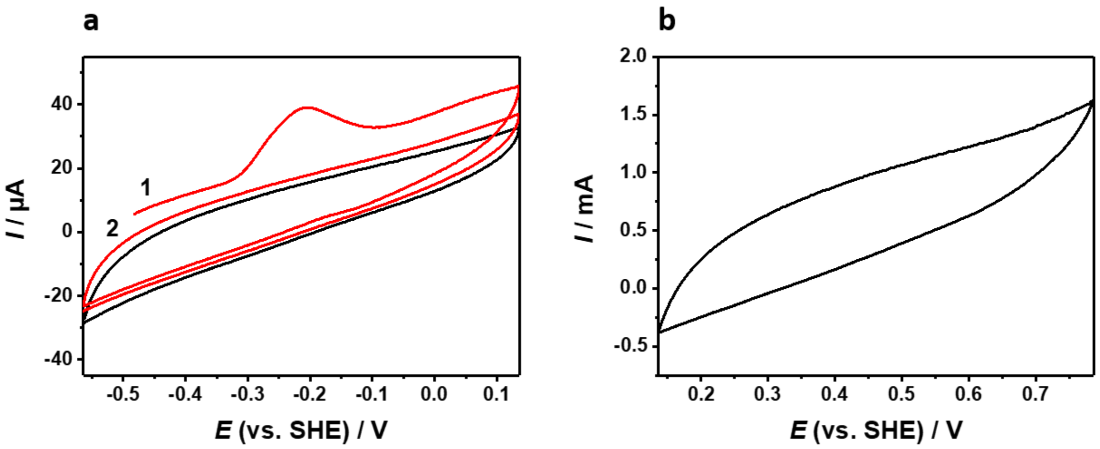

Supplementary Figure 11: Cyclic voltammograms of the (GMA-BA-PEGMA)-vio//P(N_3MA-BA-GMA)-vio/*Dv*MF-[NiFe] bioande (**a**) and the *Mv*-Box biocathode (**b**) in 0.1 M PB (pH 7.4) after the biofuel cell stability test at 0.7 V as depicted in Figure 4c and Supplementary Figure 7. **a**: red line: H_2 (first (1) and second (2)cycle), black line: argon; **b**: back line: air; all voltammograms were recorded with a scan rate of 10 mV s^{-1} and in gas breathing mode.

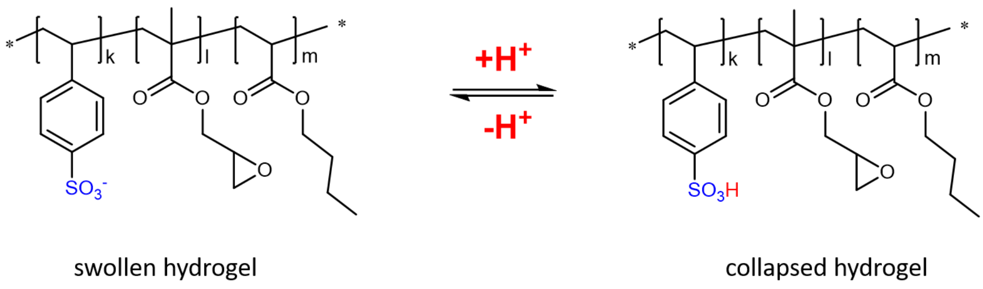

Supplementary Figure 12: Chemical structure and swollen and collapsed state of the redox silent and pH sensitive polymer P(SS-GMA-BA) that was used as stabilizing layer in the two-layer configuration of the bioanode. Under neutral and basic conditions, the sulfonate groups (blue) are fully deprotonated, and the polymer backbone reveals a highly hydrophilic nature and thus a high solubility. Under acidic conditions the sulfonate groups are protonated and the hydrogel collapses and forms a stable rather insoluble layer on top of the active redox polymer/hydrogenase layer. The nominal composition of P(SS-GMA-BA) is SS = 50 mol%, GMA = 30 mol% and BA = 20 mol %.[4]

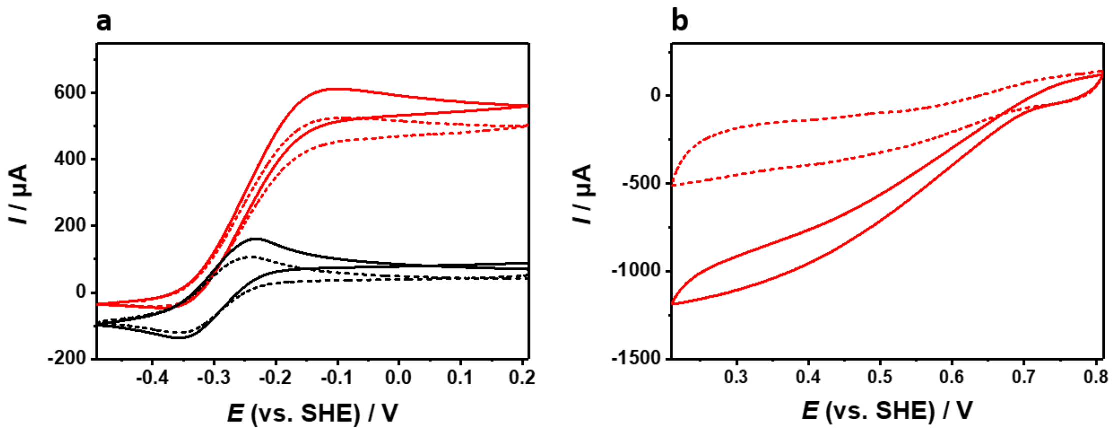

Supplementary Figure 13: Cyclic voltammetric characterization in 0.1 M phosphate buffer (pH 7.4) of the P(SS-GMA-BA) stabilized *Dv*MF-[NiFe] based bioanode (**a**) and *Mv*-Box biocathode (**b**) before (solid lines) and after (dashed lines) the long term biofuel cell measurement depicted in Figure 4 in the main text under turnover (red lines, gas breathing mode) and non-turnover conditions (black lines). The lower current amplitude of the viologen modified redox polymer under non-turnover conditions in a indicates that not only enzyme deactivation but also polymer disintegration contributes to the slight current decrease after the long-term evaluation. The heavily decreased absolute currents of the biocathode after the biofuel cell test clearly indicate that this is the limiting electrode in terms of stability. The proposed bioanode architecture including a stabilizing top polymer layer ensures a stable bioanode within the time scale of the long-term discharge. All voltammogram were recorded with a scan rate of 10 mV s^{-1}.

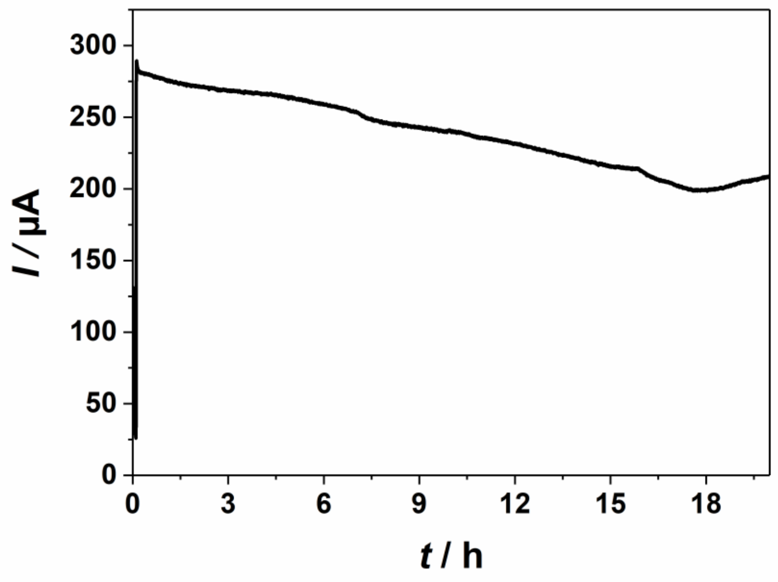

Supplementary Figure 14: Chronoamperometric experiment with a P(GMA-BA-PEGMA)-vio//P(N_3MA-BA-GMA)-vio/DvMF-[NiFe] based bioanode covered with the P(SS-GMA-BA) stabilizing layer at a applied potential of +0.16 V vs. SHE in 0.1 M phosphate buffer (pH 7.4) in gas breathing mode. The lifetime of the bioanode could be drastically enhanced by the introduction of the stabilizing polymer layer (cf. Supplementary Figure 10b, red line).

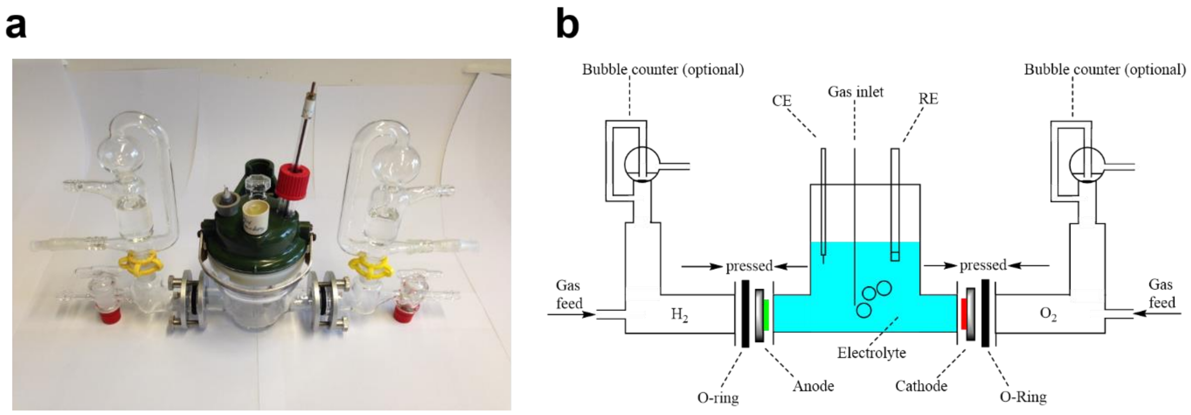

Supplementary Figure 15: Photo (**a**) and schematic (**b**) of the used custom-made gas breathing cell. Note that not only the gas diffusion layers (anode and cathode) but also an additional counter and reference electrode can be immersed into the electrolyte. The cell was specifically designed for this study and it was manufactured in the workshop of the Ruhr-University Bochum. The photo was taken by Julian Szczesny.

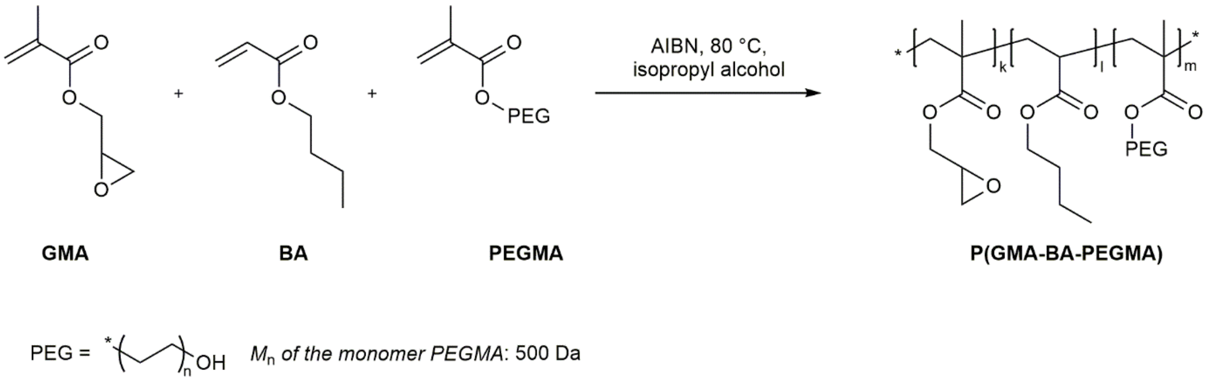

Supplementary Figure 16: Synthesis of the polymer backbone P(GMA-BA-PEGMA) via a free radical polymerization with the three comonomers GMA (glycidyl methacrylate), BA (butyl acrylate) and PEGMA (poly(ethylene glycol) methacrylate). The nominal composition of the polymer is k = 49.5 mol%, l = 42.5 mol%, m = 8 mol%, the actual composition was determined via the integral ratio of the individual monomers determined from ^{1}H-NMR experiments and was calculated to be k = 65 mol%, l = 32 mol%, m = 3 mol% (see Supplementary Figure 12).

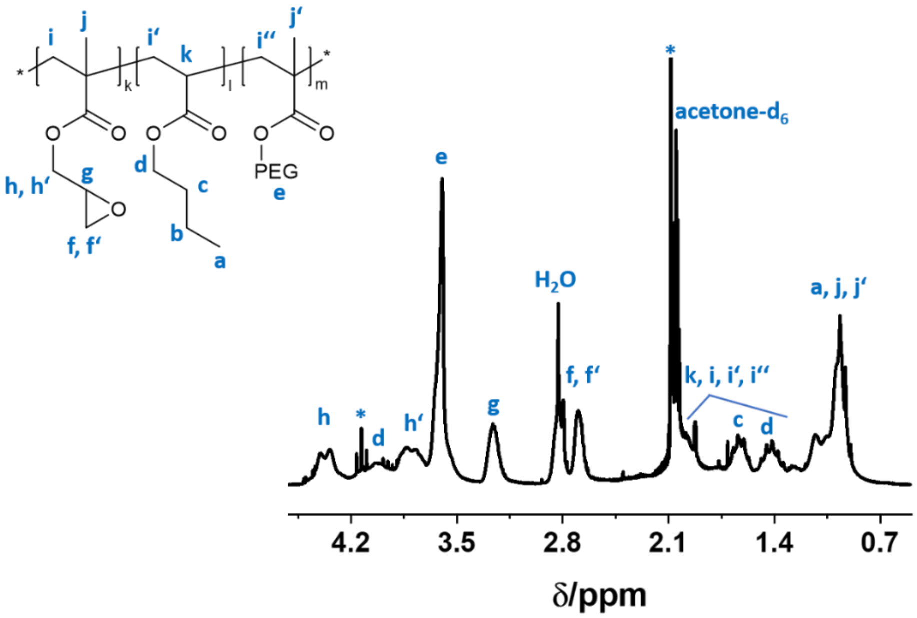

Supplementary Figure 17: ^{1}H-NMR (200.13 MHz, acetone-d_6) spectrum of the polymer back-bone P(GMA-BA-PEGMA) including signal assignment. All signals are consistent with the molecular structure of the polymer backbone. * indicates signals from residual solvents that were used during workup and sample preparation (diethyl ether and acetone).

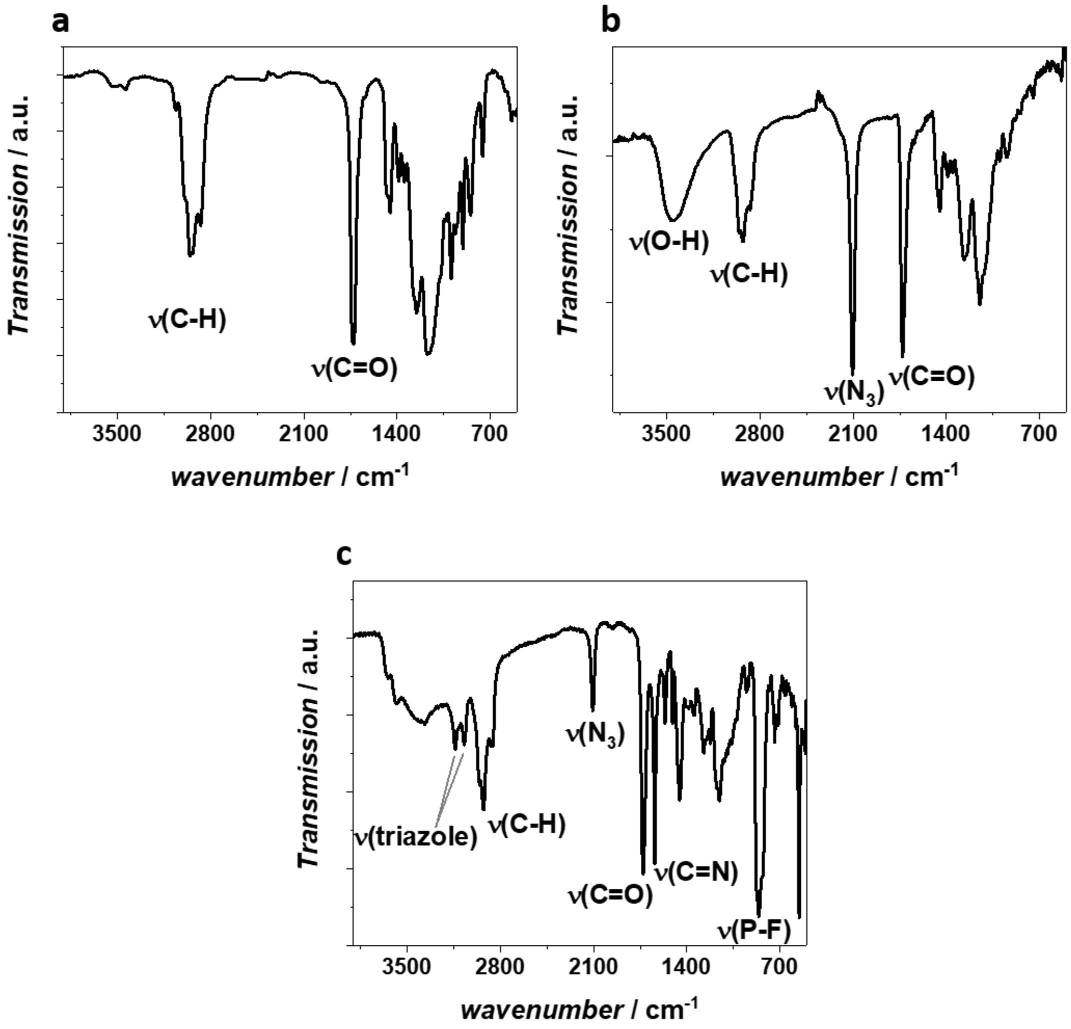

Supplementary Figure 18: Reflectance FTIR spectra of the polymer backbone(GMA-BA-PEGMA) (**a**), the azide-modified backbone P(GMA-BA-PEGMA)-N_3 (**b**) and the viologen-modified redox polymer P(GMA-BA-PEGMA)-vio (**c**). Samples were drop-coated on gold substrates from acetone solution. After modification with NaN_3 a strong band at 2104 appears that corresponds to the R-N_3 group in the polymer (cf. **a** and **b**). After the click reaction strong bands at 3135 and 3071 cm^{-1} are visible that can be assigned to the newly formed triazole group by the copper catalyzed 1,3-dipolar cycloaddition between the N_3 group and the terminal alkyne of the viologen unit (**c**).

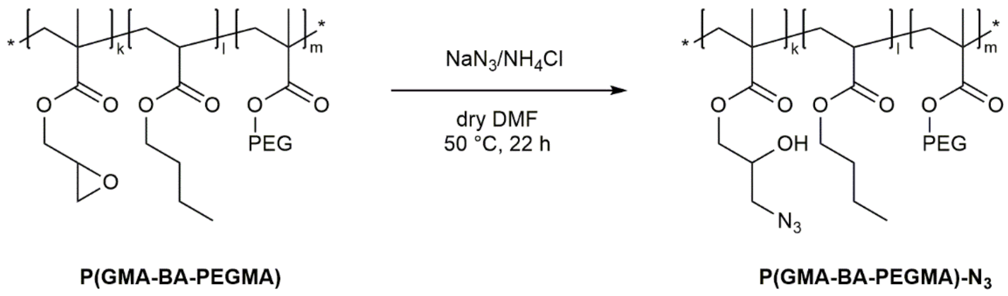

Supplementary Figure 19: Synthesis of the azide-modified polymer backbone P(GMA-BA-PEGMA)-N_3 in dry dimethyl formamide (DMF) using NaN_3 as the azide source. ^{1}H-NMR and reflectance-FTIR measurements reveal a complete conversion of the epoxide within the polymer. Composition (assuming quantitative conversion of the epoxide groups): k = 65 mol%, l = 32 mol%, m = 3 mol%.

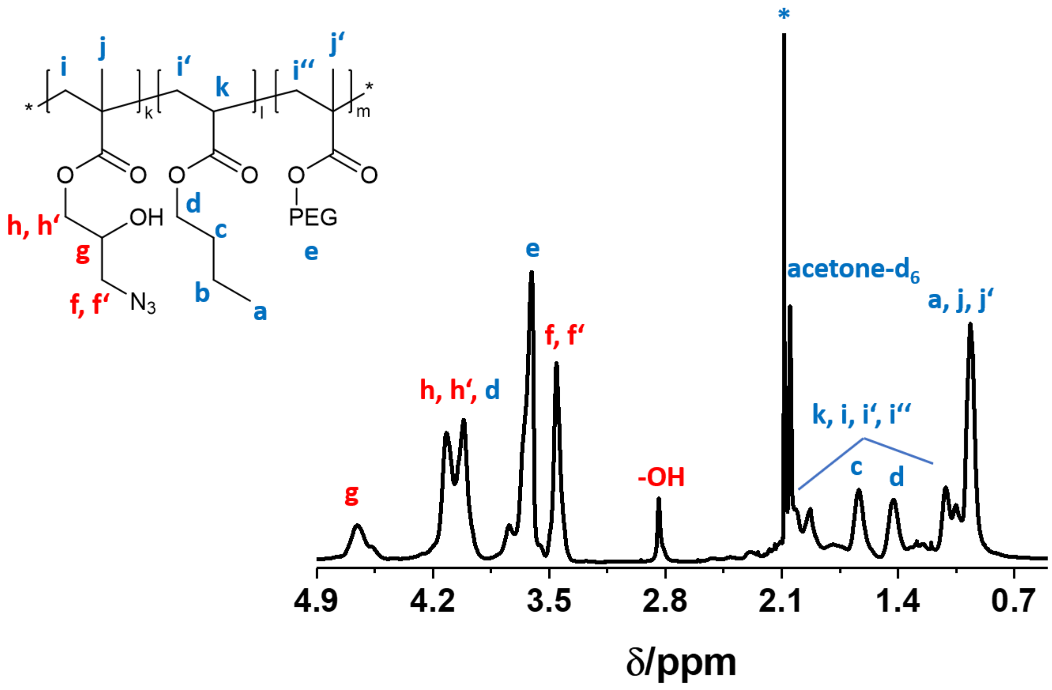

Supplementary Figure 20: ^{1}H-NMR (400.13 MHz, acetone-d_6) spectrum of the azide-modified polymer backbone P(GMA-BA-PEGMA)-N_3 including signal assignment. Signals marked in red can be assigned to the newly formed N_3-modified side chain. * indicates signals from residual solvents that were used for sample preparation (acetone).

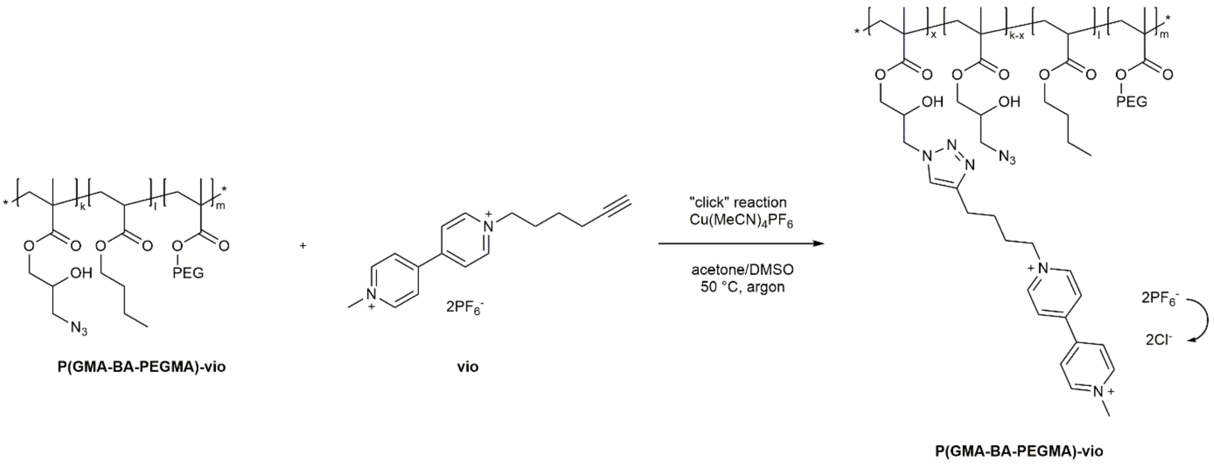

Supplementary Figure 21: Synthesis of the viologen-modified redox polymer P(GMA-BA-PEGMA)-vio. The introduction of the viologen groups to the polymer backbone was conducted by means of click chemistry using $[Cu(MeCN)_4]PF_6$ as catalyst. The hydrophobic PF_6^- counter-ions that are required to perform the reaction in organic solvents were exchanged in a meta-thesis reaction against more hydrophilic Cl^- ions. ^{1}H-NMR and reflectance-FTIR measurements reveal that some N_3 moieties still exist after modification (note that only 0.8 eq. of the alkyne-modified viologen unit were used for the modification to minimize the amount of free viologens during the purification step).

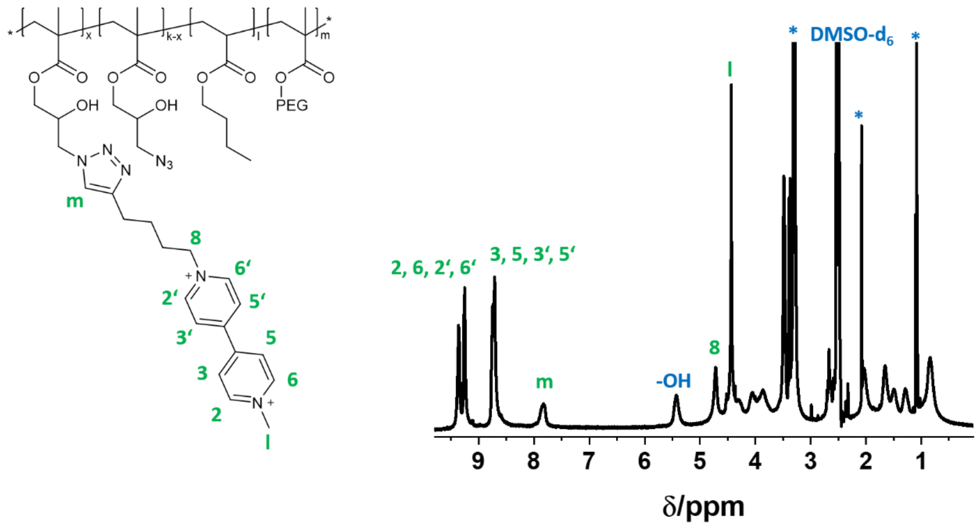

Supplementary Figure 22: ^{1}H-NMR (400.13 MHz, DMSO-d$_6$) spectrum of the viologen-modified redox polymer P(GMA-BA-PEGMA)-vio including signal assignment of characteristic protons of the viologen unit (in green). The signal at 7.82 ppm (m) is assigned to the proton f the formed triazole unit, indicating a successful product formation. Signals from the alkyl linker chain that connects the viologen with the polymer and the signals of the polymer backbone are located between 1 to 3 ppm and strongly overlapping, which hampers signal assignment. Signals of residual solvents from workup and sample preparation were marked with *.

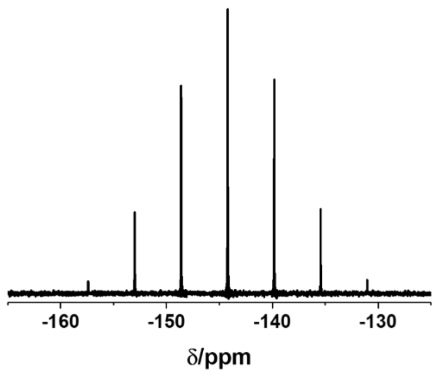

Supplementary Figure 23: ^{31}P{^{1}H}-NMR (161.98 MHz, DMSO-d$_6$) spectrum of the PF_6^- salt P(GMA-BA-PEGMA)-vio. The coupling constant for the P-F coupling was estimated to be $J_{P,F}$ = 713 Hz. The signal pattern (septet) is consistent with the molecular structure of the PF_6^- moiety.

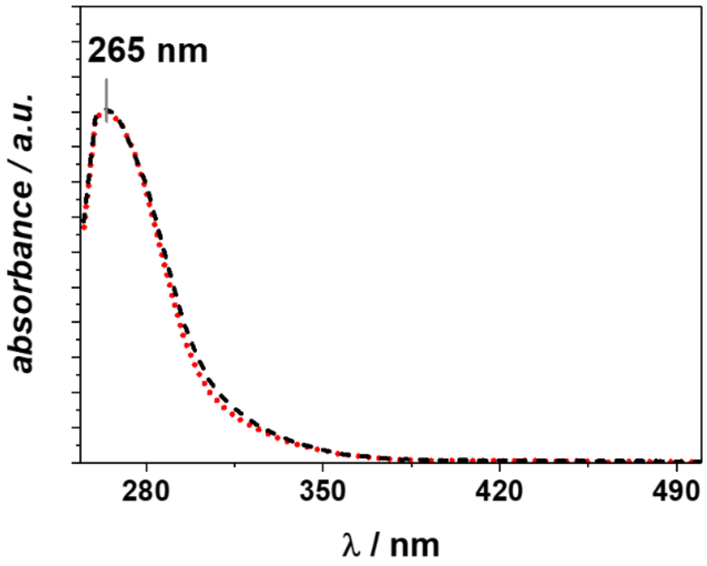

Supplementary Figure 24: UV-vis absorption spectra of the free viologen (red dotted line) and the redox polymer P(GMA-BA-PEGMA)-vio (black dashed line) in dimethyl sulfoxide. The attachment of the viologen unit to the polymer backbone has no significant effect on the electronic structure of the viologen unit since both spectra show identical absorption maxima located at 265 nm. For comparative purposes, the spectra are normalized to the absorption maxima.

Supplementary References

1. Ruff, A. *et al.* Protection and Reactivation of the [NiFeSe] Hydrogenase from Desulfovibrio vulgaris Hildenborough under Oxidative Conditions. *ACS Energy Lett.* **2,** 964–968 (2017).
2. Oughli, A.A. *et al.* A redox hydrogel protects the O_2-sensitive FeFe-hydrogenase from Chlamydomonas reinhardtii from oxidative damage. *Angew. Chem. Int. Ed.* **54,** 12329–12333 (2015).
3. Plumeré, N. *et al.* A redox hydrogel protects hydrogenase from high-potential deactivation and oxygen damage. *Nature Chem.* **6,** 822–827 (2014).
4. Teanphonkrang, S. *et al.* Tuned Amperometric Detection of Reduced β-Nicotinamide Adenine Dinucleotide by Allosteric Modulation of the Reductase Component of the p-Hydroxyphenylacetate Hydroxylase Immobilized within a Redox Polymer. *Anal. Chem.* **90,** 5703–5711 (2018).
